# Climate change adaptation in conflict-affected countries: A systematic assessment of evidence

**DOI:** 10.1007/s43621-021-00052-9

**Published:** 2021-09-27

**Authors:** A. Sitati, E. Joe, B. Pentz, C. Grayson, C. Jaime, E. Gilmore, E. Galappaththi, A. Hudson, G. Nagle Alverio, K. J. Mach, M. van Aalst, N. Simpson, P. Nayna Schwerdtle, S. Templeman, Z. Zommers, I. Ajibade, L. S. Safaee Chalkasra, P. Umunay, I. Togola, A. Khouzam, G. Scarpa, Global Adaptation Mapping Initiative Team, E. Coughlan de Perez

**Affiliations:** 1 United Nations Office for Disaster Risk Reduction (UNDRR), Geneva, Switzerland; 2 World Resources Institute, Washington, USA; 3 Department of Physical and Environmental Sciences, University of Toronto Scarborough, Scarborough, Canada; 4 International Committee of the Red Cross (ICRC), Geneva, Switzerland; 5 Faculty of Geo-Information Science and Earth Observation, University of Twente, 7514 AE Enschede, The Netherlands; 6 Red Cross Red Crescent Climate Centre, The Hague, The Netherlands; 7 Department of International Development, Community and Environment, Clark University, Worcester, MA 01610 USA; 8 Department of Geography, Virginia Tech, Blacksburg, VA USA; 9 Yale Law School, Yale University, 127 Wall St, New Haven, CT 06511 USA; 10 Oxford University Centre for the Environment, S Parks Rd, Oxford, OX1 3QY UK; 11 Nicholas School of the Environment at Duke University, Sanford School of Public Policy at Duke University, Duke University School of Law, 9 Circuit Dr, Durham, NC 27701 USA; 12 Department of Environmental Science and Policy, Rosenstiel School of Marine and Atmospheric Science, University of Miami, Miami, FL USA; 13 Leonard and Jayne Abess Center for Ecosystem Science and Policy, University of Miami, Coral Gables, FL USA; 14 International Research Institute for Climate and Society, Columbia University, New York, USA; 15 African Climate and Development Initiative, University of Cape Town, Cape Town, South Africa; 16 Faculty of Medicine and University Hospital, Heidelberg Institute of Global Health (HIGH), Heidelberg University, Heidelberg, Germany; 17 Monash Nursing and Midwifery, Faculty of Medicine, Nursing and Health Sciences. Monash University, Clayton, Australia; 18 Columbia University, New York, USA; 19 United Nations Office for the Coordination of Humanitarian Affairs (UNOCHA), New York, USA; 20 Department of Geography, Portland State University, 1721 SW Broadway, Portland, OR 97201 USA; 21 Department of Geography, Environment and Geomatics, University of Ottawa, Simard Hall, Rm 047, Ottawa, ON K1N 6N5 Canada; 22 International Development Research Centre, 150 Kent St., Ottawa, ON K1P 0B2 Canada; 23 Nature for Climate Branch, Ecosystems Division, UNEP, Nairobi, Kenya; 24 Yale School of Environment, 360 Prospect Street, New Haven, CT 06511 USA; 25 Mali-Folke Centre, Bamako, Mali; 26 University of Leeds, Leeds, UK; 27 Friedman School of Nutrition Science and Policy, Tufts University, Medford, USA

## Abstract

People affected by conflict are particularly vulnerable to climate shocks and climate change, yet little is known about climate change adaptation in fragile contexts. While climate events are one of the many contributing drivers of conflict, feedback from conflict increases vulnerability, thereby creating conditions for a vicious cycle of conflict. In this study, we carry out a systematic review of peer-reviewed literature, taking from the Global Adaptation Mapping Initiative (GAMI) dataset to documenting climate change adaptation occurring in 15 conflict-affected countries and compare the findings with records of climate adaptation finance flows and climate-related disasters in each country. Academic literature is sparse for most conflict-affected countries, and available studies tend to have a narrow focus, particularly on agriculture-related adaptation in rural contexts and adaptation by low-income actors. In contrast, multilateral and bilateral funding for climate change adaptation addresses a greater diversity of adaptation needs, including water systems, humanitarian programming, and urban areas. Even among the conflict-affected countries selected, we find disparity, with several countries being the focus of substantial research and funding, and others seeing little to none. Results indicate that people in conflict-affected contexts are adapting to climate change, but there is a pressing need for diverse scholarship across various sectors that documents a broader range of adaptation types and their results.

## Introduction

People living in conflict-affected areas are more vulnerable to climate-related impacts and extreme events, and they have fewer resources to respond, mitigate or recover from those impacts [33, 43]. State services, including health services, food systems, and early warning systems, are often lacking or unreliable in these regions [13], heightening the sensitivity of people to hazards while simultaneously impeding risk reduction and recovery capacity. Additionally, those exposed at the intersection of climate and conflict can lack land tenure and experience frequent displacement [46, 47]. During conflicts, key assets are destroyed, including personal belongings, critical infrastructure, and environmental services [45, 64]. Farming and livelihoods can be disrupted, especially when movement restrictions are in place [54, 79]. Furthermore, economic opportunities in conflict-affected regions are limited, resulting in few options for livelihood diversification [36].

In addition to these external barriers limiting adaptation and coping mechanisms, people living in conflict-affected areas have a variety of identity-based vulnerabilities which exacerbate the effects of climate change in such areas. For instance, women and men are differentially impacted by climate change due to persistent inequalities and the socialization of gender roles which hinder and affect women’s adaptation and coping mechanisms. Women carry a disproportionate burden in conflicts when it undermines their asset base, including productive capacity and other income-generating activities [35]. Moreover, people can have physical and cognitive disabilities resulting from the conflict itself, which can intersect with marginalization based on gender and ethnicity that can further be compounded by failed structures of governance [16].

These intersecting vulnerabilities and climate-related challenges can have compounding and catastrophic impacts in conflict-affected areas [65, 76]. Despite these fragile contexts, people living in these conflict-affected areas are still coping and adapting to climate change, although there is often little support from private sources, national or international institutions. Donors are risk-averse to making large contributions of adaptation finance in conflict-affected zones due to the unpredictable circumstances, and instead tend to prioritize adaptation funding for countries and regions with stable governance [32, 48, 72]. Additionally, climate adaptation research contains a strong bias towards stable and easy-to-reach locations [3, 30].

In conflict-affected areas, immediate needs, protection, peacebuilding, and stabilization actions are prioritized, while responsibilities for promoting disaster risk reduction (DRR) are frequently neglected [61, 70]. Acting ahead of predictable climate-related hazards is often not a priority [70] and conflict-affected states have difficulty implementing basic early warning systems (EWS) [28]. Additionally, although enhancing responses such as EWS has been included in DRR strategies of countries such as Afghanistan, Iraq, and Myanmar [8, 27, 50], there is no robust evidence about implementation, effectiveness, and lessons learnt.

There is substantial literature confirming climate events as one of many contributing drivers of conflict [26, 41, 71]. For example, climate change increases conflict risk through economic shocks and natural resource dependency, but potentially even more troubling is the inverse: that conflict increases vulnerability and worsens the impacts related to climate variability and change. Adaptation programmes themselves, in aiming to reduce vulnerability for one group in a complex situation, can worsen the situation for another group and incite further conflict [1].

Even though people in conflict-affected areas are some of the most vulnerable to climate change, there is evidence that these regions tend to receive less investment for climate change adaptation [59]. This paper sets out to systematically analyze what climate change adaptation is happening in conflict-affected areas despite such challenging contexts. Starting with the published literature, a systematic literature review analyzes adaptation activities that are documented in 15 conflict-affected countries. This database of peer-reviewed literature is then compared with adaptation policies and funding streams for climate change adaptation in each country, to identify areas of investment or gaps.

In conducting this systematic literature review, we seek to determine what scholarly research exists on climate change adaptation in conflict-affected contexts. We identify a series of gaps in the research by comparing this database to the policy priorities and funding investments in each conflict-affected country, as well as to average results in non-conflict-affected countries. We also characterize climate funding flows in each country to identify areas of implementation and places for further progress.

## Methodology

We define conflict as an aggressive interaction between groups of people who perceive agreement (or disagreement) on political ideologies (or interest) resulting from natural resources, sectarian, or outsider interference, which involves armed responses [54, 71]. To identify a set of representative countries for this review, we included 15 countries that have experienced protracted conflicts in recent decades and which have the largest operations by the International Committee of the Red Cross (ICRC) between 2015 and 2020 [33]. As shown in Table 1, the set of conflicts constituting our sample countries represent a diversity of conflict types, from civil war in Afghanistan to sectarian conflict in Nigeria. For context, we include the number of events of organized violence per country between 2010–2020, as recorded in the Uppsala Georeferenced Event Dataset [62] and the population estimates for each country based on the 2020 World Bank statistics. Each of these 15 countries was in the top 34 of all countries in terms of the number of conflict events during this time.

**Table 1:** Overview of the diversity of the conflict situations in each of the 15 countries included in this study, with the number of conflict events on record for each country

Country name	Number of conflict events 2010–2020	2020 Population estimates (World Bank^a^)	Provinces with more than 20 conflicts 2010–2020	Type of conflict
Afghanistan	23,936	38,928,340	Badakhshan, Badghis, Baghlan, Balkh, Fara, Faryab, Ghazni, Ghor, Hilmand, Hirat, Jawzjan, Kabul, Kandahar, Kapisa, Khost, Kunar, Kunduz, Laghman, Logar, Nangarhar, Nimroz, Nuristan, Paktika, Paktya, Parwan, Samangan, Sari Pul, Takhar, Uruzgan, Wardak, Zabul	Civil War
Central African Republic	1254	4,829,760	Bangui, Basse-Kotto, Haute-Kotto, Haut-Mbomou, Kémo, Lobaye, Mambéré-Kadéi, Mbomou, Nana-Grébizi, Nana-Mambéré, Ombella-M'Poko, Ouaka, Ouham, Sangha-Mbaéré, Vakaga	Sectarian
Democratic Republic of the Congo	2282	89,561,400	Haut-Uélé, Ituri, Kasaï Occidental, Kasaï Oriental, Kasaï, Kasaï-Central, Katanga, Kinshasa city, Kinshasa, Kongo Central, Kwilu, Lomami, Lualaba, Mai-Ndombe, Maniema, Nord Kivu, Orientale, Sud Kivu, Tanganyika	Political instability
Iraq	4420	40,222,500	Al Anbār, Al Başrah, Al Muthanná, Al Qādisīyah, An Najaf, Arbīl, As Sulaymānīyah, Bābil, Baghdād, Dahūk, Dhī Qār, Diyālá, Karbalā’, Kirkūk, Maysān, Nīnawá, Şalāḩ ad Dīn, Wāsiţ	Civil War
Israel, West Bank and Gaza strip	509	9,216,900	Gaza Strip, Southern district, West Bank	Territorial Dispute
Lebanon	118	6,825,440	Baalbek-Hermel, North Lebanon	Political instability
Libya	1103	6,871,290	Al Wahat, Benghazi, Darnah, Jabal al Gharbi, Misrata, Mourzouq, Sabha, Surt, Tripoli, Zawiyah	Civil War
Mali	795	20,250,830	Gao, Kidal, Ménaka, Mopti, Segou, Tombouctou	Transnational Terrorism
Myanmar	1027	54,409,790	Chin, Kachin, Kayin, Rakhine, Shan	Sectarian
Nigeria	4079	206,139,590	Adamawa, Bauchi, Benue, Borno, Ekiti, Gombe, Kaduna, Kano, Kogi, Lagos, Nasarawa, Ogun, Plateau, Rivers, Taraba, Yobe	Sectarian
Somalia	3714	15,893,220	Banaadir, Lower Shabelle, Lower Juba, Bay, Gedo, Hiran, Middle Shabelle, Bakool, Galgudud, Middle Juba, Mudug	Transnational Terrorism
South Sudan	802	11,193,730	Unity, Central Equatoria, Jonglei, Upper Nile, West Bahr-al-Ghazal, Lakes, West Equatoria, Warap	Civil War
Syrian Arab Republic	59,312	17,500,660	Al Hasakah, Aleppo, Ar Raqqah, As Suwayda, Damascus, Daraa, Deir ez Zor, Hamah, Homs, Idlib, Latakia, Quneitra, Rif Dimashq, Tartus	Civil War
Ukraine	1002	44,134,690	Donetsk Oblast, Luhansk Oblast	Territorial Dispute
Yemen	2422	29,825,970	Abyan, Ta'izz, al-Bayḑā’, ‘Amrān, al-Ḩudaydah, Ḩaḑramawt, Shabwah, Amānat al ‘Āşimah, Ma'rib, Şa‘dah, Ḩajjah, Laḩij, aḑ-Ḑāli‘, al-Jawf, Şan‘ā’, Ibb	Civil War

To provide context for the reader on the geographical scope, we also list the locations of large numbers of conflict events [62, 67], and the type of conflict occurring [66]

^a^The World Bank Population Statistics: https://data.worldbank.org/indicator/SP.POP.TOTL

To document the state of adaptation in each country, we analyze all articles for each country contained in the Global Adaptation Mapping Initiative (GAMI) dataset [11]. The GAMI dataset is a systematic collection of all peer-reviewed literature documenting climate change adaptation, carried out in three steps. GAMI follows the guidelines for systematic evidence synthesis using the ROSES established reporting standards.

The first step in the GAMI systematic literature review was creating the dataset through a keyword search of three databases: Scopus, Web of Science, and Google Scholar for all articles that included concepts of climate change and adaptation or adaptation-related responses published between 2013–2019. The terms “resilience” or “risk management” were also included to expand the concept of adaptation to all relevant literature. This yielded approximately 50,000 documents associated with, but not limited to the topic of ‘climate change adaptation’. A sample of articles were then screened by two independent (blinded) reviewers. First, the articles were screened by title and abstract and then in full text. Articles were included or excluded based on the criteria in Table 2.

**Table 2:** Inclusion and exclusion criteria for country selection

Concept	Inclusion	Exclusion
Population	Any population	No geographic or social exceptions
Exposure	Climate change	No exposure to climate change, climate variability or environmental change
Outcome	Reports on what people think and do Reports on how people respond to environmental change Responses relate to adaptation Tangible/observed behavioral responses (actions, practices, improved knowledge, altered social structure) Responses arguably reduce risk or improve adaptive capacity to climate change	Reports on biological or ecological processes Limited to assessment or vulnerability or impacts (not how people respond) Responses relate to mitigation only Responses are planned or recommended (not actual/observed) Responses do not arguably reduce risk or improve adaptive capacity to climate change
Type of study	Empirical data included—from observation or experience Systematic literature review	No empirical data included Empirical data theoretical or simulated
Time frame	Published between 2013–2019 Responses are recent	Published outside this timeframe Responses are historical
Language	Articles indexed in English, even if written in another language	

To efficiently sift through the 50,000 articles from the keyword search, a machine learning classifier was trained based on the results from an initial set of papers screened by humans. The algorithm then assigned each article a probability of being relevant, and high probability documents were all manually screened by humans. The result was 4300 articles screened manually (Fischer et al. submitted).

This yielded 1682 articles that were “screened in” because they documented observed adaptation around the world. These articles were then “coded” by two independent coders to enhance the reliability of the codes from each article (Lesnikowski et al. submitted). SysRev, an online systematic literature review application, was used for coding and data extraction. Coders with more than 10% of missing entries were marked as “unreliable”, and every article was coded by at least two “reliable” coders. For each category, the results from multiple coders were accepted when not mutually exclusive (Siders et al. submitted).

Our study makes use of two codes from this step in the process: the countries of focus for each article and relevant sectors. For this analysis, we included all GAMI articles that were tagged with one of the 15 conflict-affected countries selected above. For each country, we did an additional literature search to determine whether there were any papers that had been missed by the above protocols. This revealed two additional papers for Ukraine. This validates that the GAMI database was comprehensive, with few oversights. We also documented the countries of the affiliations of all authors of all the papers for these countries.

The GAMI systematic literature review included only scientific articles. To provide context on the priorities and adaptation actions in each country that might not be documented in the scholarly literature, we analyzed three additional sources of information for each of the 15 countries.

First, we collected records of climate finance flows to each country. We aggregated the list of all projects and their amounts from multilateral climate change adaptation funds that have been pledged to each of the 15 countries until 2019 [52]. We complemented this by analyzing the bilateral funding flows that are listed as climate change adaptation. We included all bilateral projects that have a marker as “principal” or “significant” relation to Climate Change Adaptation (CCA) [53].

Second, we documented extreme climate-related events that have happened in each country between 2010–2020. We downloaded all recorded events in the GLIDE database for each country and excluded all events and epidemics that are not potentially climate-related (e.g. COVID-19, volcanic eruptions). We relied on the GLIDE database due to its robustness and less confusion in identification of the disaster in countries with many disaster events.

We read and reviewed the contents of the journal articles, the titles and funding amounts of the adaptation flows, the description of recent extreme events, and the objectives listed in each of the policy documents. Based on these descriptive results, we highlight overall trends using descriptive statistics as well as discrepancies between the datasets in terms of content, focus, and availability of information on different sectors, populations, and hazards.

The main limitation for this study is that the systematic review undertaken through GAMI only targeted scientific articles but none from the grey literature which often have the potential to provide valuable and context-specific information/data on adaptation across the target countries. However, including such literature would most likely lead to inconsistent assessment of the various aspects of adaptation which are compounded by varying interests [21]. The breadth of GAMI’s database is extensive in terms of the number of articles that were identified, screened and coded, and this highlights its potential value to capture the breadth of the literature on adaptation [19, 22]. For instance, Asia recorded the highest number of adaptation articles identified by GAMI (34%), followed by Africa (32%) [11]. Nonetheless, a key limitation emerging from this extensive GAMI database is the publication bias particularly based on language. Evidently, there is dominance of English-language publications as compared to publications in other languages, and this makes it difficult to distinguish whether absence of adaptation reporting is a reflection of reporting in the peer-reviewed literature or lack of adaptation activities [10]. Nevertheless, the findings from this study will be easily translatable for expert-driven processes such as IPCC because the focus on peer-reviewed literature aligns well with the assessment needs.

## Results

In this section, we present an overview of the GAMI literature results on current adaptation-related activities in 15 conflict-affected countries. We then provide context on the current state of adaptation in each country through an overview of climate finance flows, climate-related disasters, and national adaptation policies.

### Literature

**Fig. 1:**
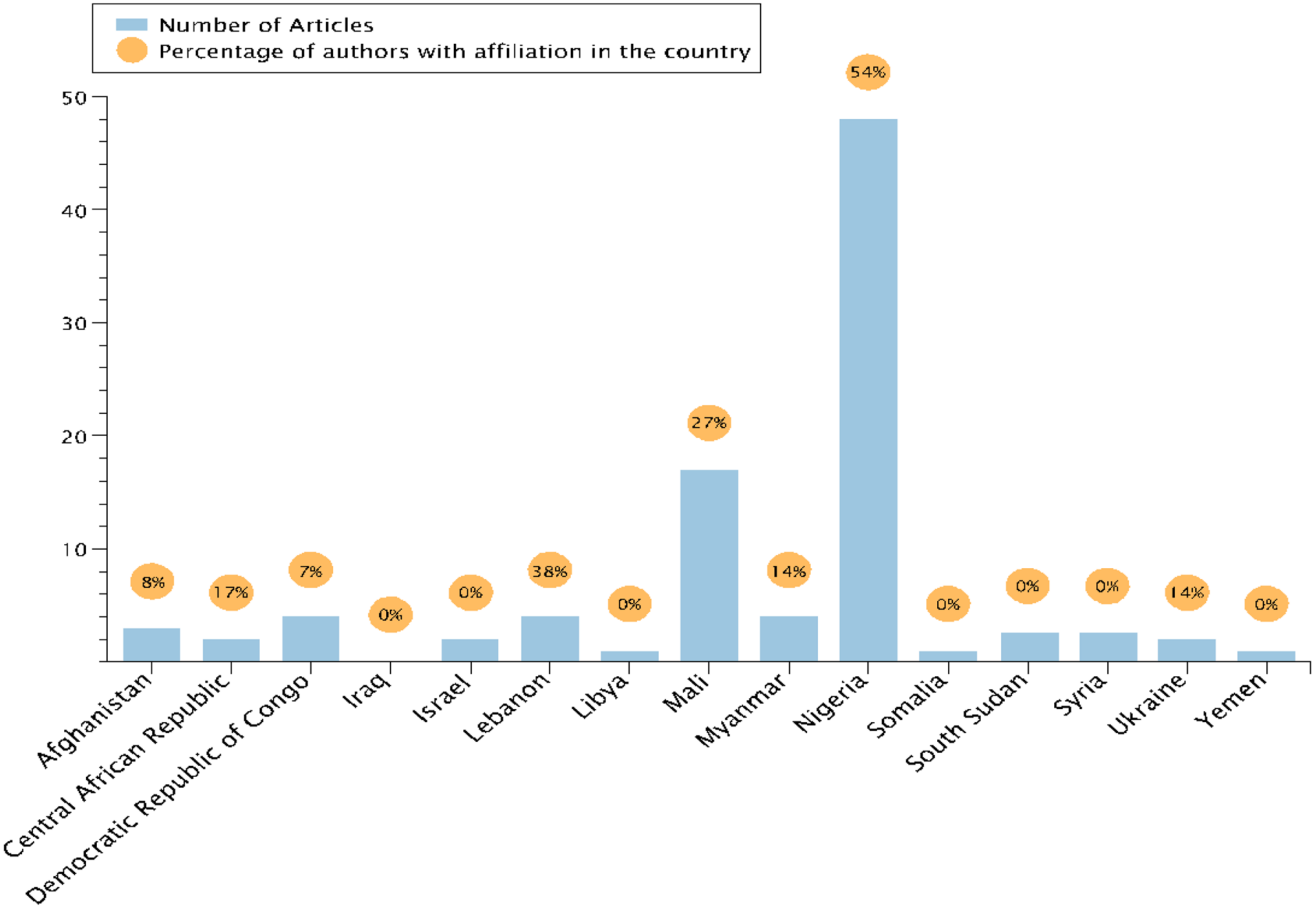
Number of articles per country in the GAMI database. Of each set of articles per country, the articles with at least one coauthor from that country are represented in orange

Documentation of observed adaptation in published articles is relatively sparse for the 15 countries included in this study. Globally, the average number of articles per country in the GAMI dataset is 13. In the 15 countries studied here, only Mali and Nigeria exceed this amount. The remaining 13 conflict-affected countries are the focus of 4 articles or fewer in the GAMI database (Fig. 1).

In addition, most of these articles do not have a single coauthor from the country being studied. Out of the 313 co-authors of all papers included in this study, only 115 have at least one affiliation in the country they are researching (Fig. 1).

**Fig. 2:**
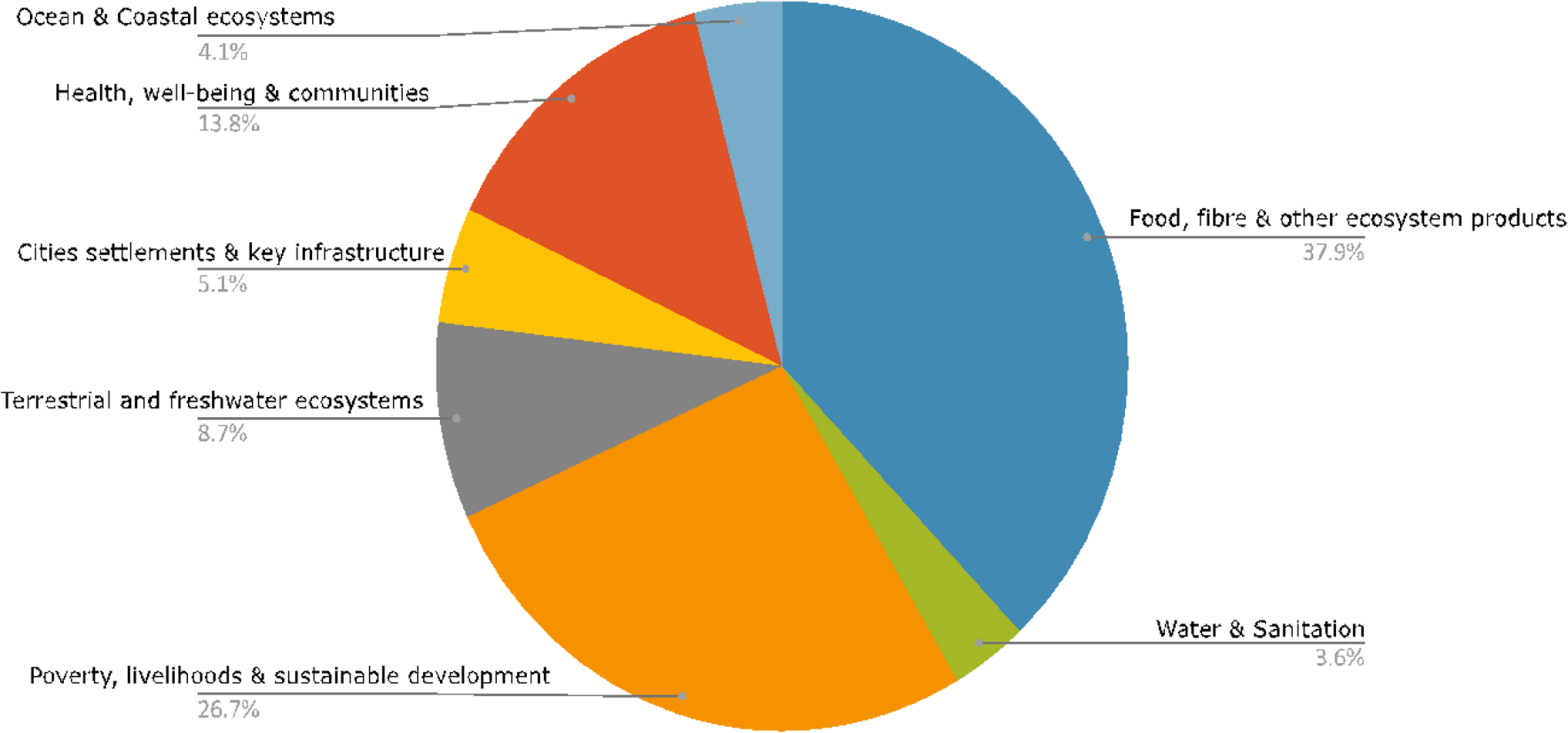
Relative frequency of sectoral focus for all papers for the 15 conflict-affected countries

Each paper was coded for its sectoral focus, with multiple choices possible for any one article. 37.9% of all articles coded were related to “Food, fibre, and other ecosystem products”. The total relative distribution of sectors across all papers is shown in Fig. 2. Very few papers focused on “Water and sanitation” or “Cities, settlements and key infrastructure”.

Among the academic literature on adaptation in conflict-affected countries, the most common sector is “Food, fibre, and other ecosystem products”. The bulk of articles tagged with this category pertained to adaptation in agricultural systems, especially smallholder agriculture. This included articles on the impact of agricultural interventions and farmer perceptions in Afghanistan [34, 37], and determining adaptation implementation and perception among farmers in Myanmar [55, 68]. There were a few studies comparing farmer adaptation across countries, for example between Lebanon and Germany [19] and between Central African Republic and Kenya [51]. For both Mali and Nigeria, more than half of the articles for each country related to the food sector, ranging from smallholder farmers in Mali [57], to articles on specific drought-tolerant crops [77] and farmers of fruits and vegetables in Nigeria [4]. In addition to crops, there were several articles in this category on fishing and aquaculture [7, 9, 49] as well as livestock [15, 17, 24, 49].

Many of the articles were tagged both with the “Food” sector as well as the “Poverty, livelihoods, and sustainable development” sector. This included articles on farmers and livestock keepers [17, 56]. The only article about adaptation in Libya was in this category, which focused on the use of traditional ecological knowledge for adaptation by the Kel Tadrart Tuareg [12]. Articles in this category often positioned climate change adaptation in the context of wider development goals and migration [6, 75].

Articles related to terrestrial and freshwater ecosystems included articles on the implementation of REDD + in the Democratic Republic of Congo and the Central African Republic [14] as well as the implications of conservation for water birds in Israel [23]. Several articles focused on human management of ecosystem services, such as community water management under climate change in Mali [18].

The category of “Health, well-being, and communities” included a diversity of impacts and adaptations. This ranged from the only article about Somalia, focused on the famine of 2011 [44], to flood management and waste management in Mali [18]. The only article on heat in cities was a global study that included one example of the use of urban greenery in Tel Aviv, Israel [31].

The “Cities settlements and key infrastructure” category also included a global article on urban adaptation; this article had a case study on the allocation of municipal budget for disaster risk reduction in Beirut, Lebanon [38]. Several articles on Nigeria in the “Cities” category include adaptations for flood management [21, 39]. There is one article on adaptation in urban and peri-urban agriculture in sub-Saharan Africa [40].

Nigeria was the only country that had articles tagged with the “Ocean and coastal ecosystems” category; these three articles focused on adaptation in fisheries and food security in the rural poor [9, 20, 49].

Articles that included a focus on water and sanitation focused both on drought and flooding, including Iqbal et al. [34] in Afghanistan, and Nguimalet [51] in Central African Republic. Heikkila et al. [29] looked at adaptation as governed by the Mekong River Commission, including in Myanmar.

### Funding flows

**Fig. 3:**
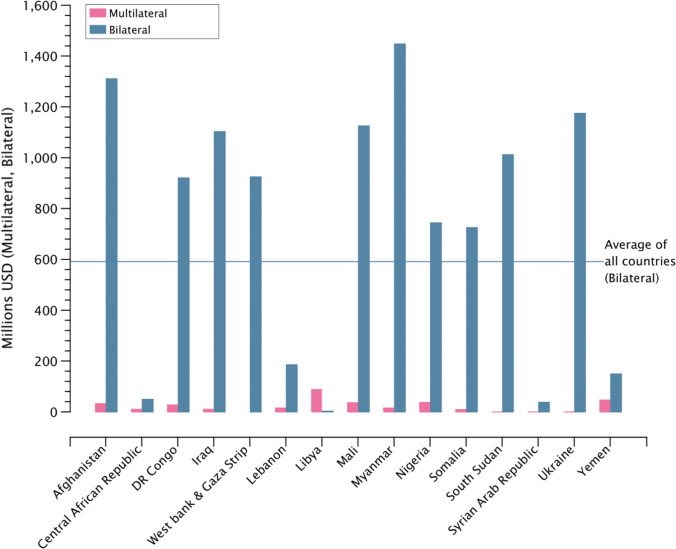
Funding amounts pledged via multilateral and bilateral climate change adaptation funds

The total amount of funding that has been pledged in multilateral climate funds as well as bilateral aid per country is depicted in Fig. 3. While this analysis does not seek to statistically analyze all the factors that contribute to the determination of funding amounts per country (see Weiler [72], others), it is clear that many of these conflict-affected countries are receiving funding that can be used for climate change adaptation. The amounts vary dramatically between countries, with several countries receiving consistently more than the world average (Afghanistan, Mali), while other countries receive significantly less (Syria, Lebanon, and CAR).

The focus of funding flows also differs between countries. Table 3 shows the sector receiving the highest amount of bilateral funding with a primary or significant objective of climate change adaptation. This varies from “Agriculture” in Afghanistan, Mali, and Myanmar, to “Emergency response” in CAR, South Sudan, and Syria, to “Water” in Iraq, Lebanon, Ukraine, the West Bank and Gaza Strip, and Yemen.

**Table 3:** Sector receiving the highest amount of pledged bilateral funding that has been tagged with climate change adaptation as a “primary” or “secondary” objective, between 2010–2019

Country	Sector with highest bilateral funding
Afghanistan	Agriculture
Central African Republic	Emergency response
DR Congo	Environment protection
Iraq	Water
Lebanon	Water
Libya	Fishing
Mali	Agriculture
Myanmar	Agriculture
Nigeria	Other social infrastructure & services
Somalia	Government & civil society
South Sudan	Emergency response
Syria	Emergency response
Ukraine	Water
West Bank and Gaza Strip	Water
Yemen	Water

However, many of the conflicts in these 15 countries are localized in specific regions or provinces. Accordingly, funding flows for climate change adaptation projects in these regions often focus sub-nationally on specific regions for specific objectives. In many cases, these projects are not spread equally throughout the country, such as the case of Iraq (Table 3), where multilateral climate change adaptation projects are concentrated in districts with fewer conflicts. However, most countries in this study do not see a bias as clear as Iraq, with projects more evenly spread between the more conflict-affected and less conflict-affected regions, such as in the case of Mali (Table 4).

**Table 4:** Number of conflicts per sub-national region in Iraq and Mali between 2010–2019, and number of multilateral climate change adaptation projects in each sub-national region during that same time period

IRAQ	Number of conflicts	Multilateral CCA projects	MALI	Number of conflicts	Multilateral CCA projects
Nīnawá province	1,332		Mopti region	321	5
Baghdād province	791		Gao region	117	1
Al Anbār province	769		Kidal region	105	1
Şalāḩ ad Dīn province	540		Tombouctou region	97	1
Diyālá province	355		Ménaka region	83	
Kirkūk province	312		Segou region	45	4
Arbīl province	90		Koulikoro region	12	5
Bābil province	70		Bamako region	9	1
Dahūk province	65		Sikasso region	4	4
Karbalā’ province	26		Kayes region	2	7
Wāsiţ province	26				
Al Başrah province	14				
Dhī Qār province	8	1			
As Sulaymānīyah province	7				
An Najaf province	6				
Al Muthanná province	3	1			
Al Qādisīyah province	3	1			
Maysān province	3	1			

Analysis of the project documents of several climate change adaptation projects reveals that adaptation intervention is affected by the extant security situation in three separate ways. Firstly, through the criteria for selecting the target site of intervention, where importance is given to the security situation of the site. Secondly, through the project risk management strategy protocol wherein if the security situation were to deteriorate, a different site would be chosen for adaptation intervention. Thirdly, the reduced stakeholder engagement due to a fragile security environment could affect equity considerations during the project planning phases. This means that by design, relatively secure areas in conflict prone regions could attract more adaptation intervention funding than other regions.

### Disasters

**Fig. 4:**
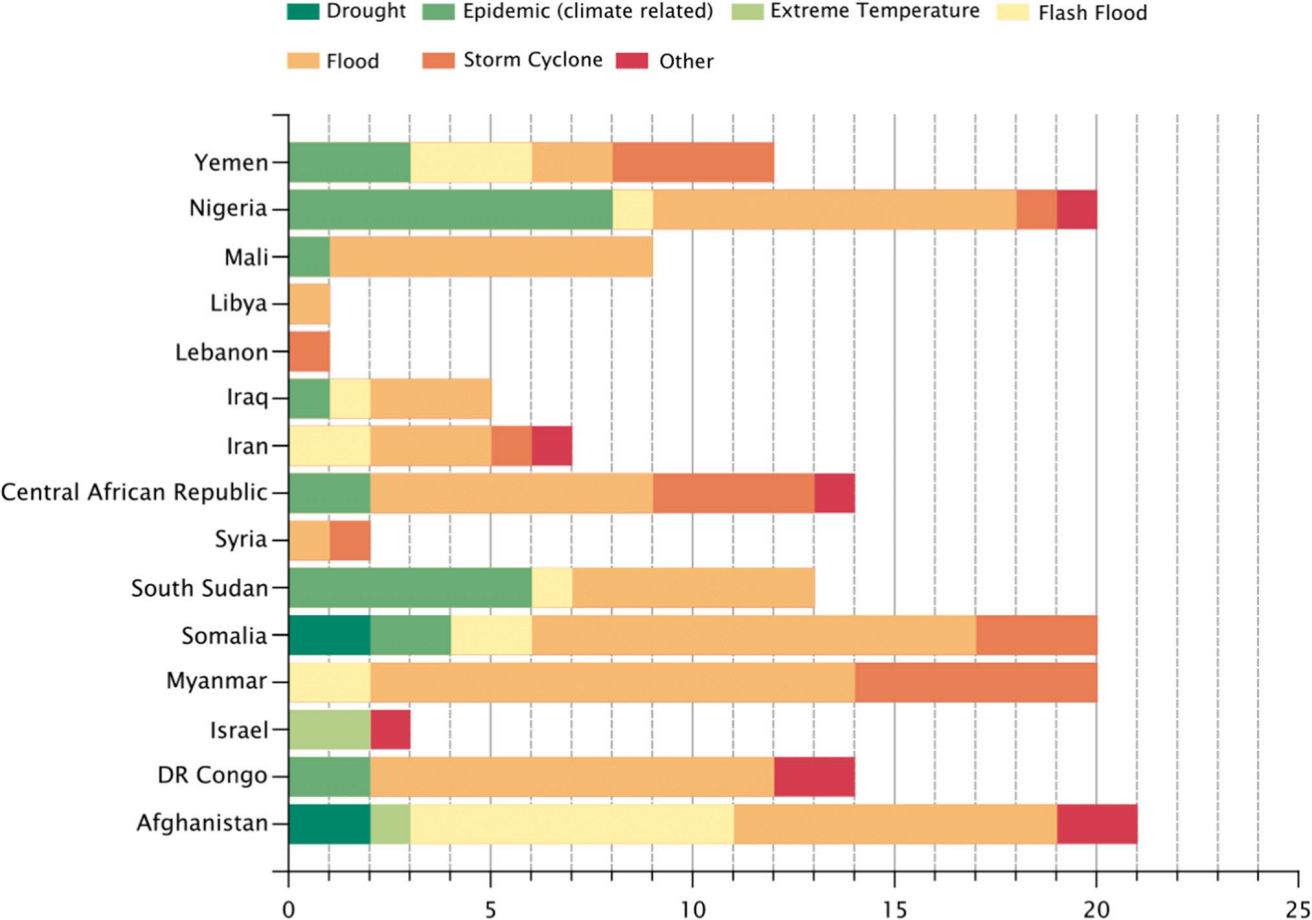
Frequency of climate-related disaster events between 2010–2019 per country (GLIDE)

While people living in these 15 conflict-affected countries are managing multiple risks related to the respective conflicts, there have also been a number of climate-related disasters recorded in each country during the study’s focal period. Figure 4 depicts the recorded climate-related disaster events per country; floods and epidemics have been recorded frequently across most countries between 2010–2019. This analysis is included to illustrate the diversity of climate-related hazards that are affecting these countries, as a simple point of comparison with the scope of the academic literature on climate change adaptation.

## Analysis and discussion

Climate change adaptation is occurring around the world in places that are simultaneously affected by armed conflicts. We review and summarize the recent literature on climate change adaptation efforts and conflict-affected areas. These regions have experienced various climate-related disasters in the last decade, such as unexpected drought, floods, extreme weather, and disease conditions. Both bilateral and multilateral funding targeting climate change adaptation is reaching many conflict-affected areas.

Regions have experienced various climate-related disasters in the last decade, and there is evidence of coping and adaptation strategies. For example, agricultural adjustments, including changing crop varieties and planting times in Afghanistan [37] and changing production and marketing strategies for livestock in Mali [24]. Indigenous knowledge has also been applied to enable adaptation, for example in Libya to deal with erratic rainfall [12].

Seasonal and permanent migration were also frequently cited adaptation strategies. For instance, Afghan farmers sought temporary work due to climate variability [34], and similar experiences were found in CAR and Yemen [51, 75].Rural–urban migration was reported in the Democratic Republic of the Congo [28]. Additionally, migration was a primary coping strategy during the 2011 drought in Somalia [44].

Climate adaptation research towards sectoral development in conflict-affected countries shows a large knowledge gap. Most papers are focused on adaptation in the agricultural sector, especially by smallholder farmers and livestock keepers. These papers were classified in Fig. 2 as related to “Food, fibre, and other ecosystem products” as well as “Poverty, livelihoods, and sustainable development”. However, adaptation is also needed in the sectors under-represented in the literature, such as urban areas, health, and infrastructure. Literature on the gender dimension of climate adaptation in conflict-affected countries is also limited.

There is only one study in the GAMI database for any of these 15 countries that is focused on urban issues. Johnson and Blackburn [38] include a case study on how the government of Beirut, Lebanon, has allocated municipal budget funds to disaster risk reduction. The lack of information on adaptation in urban areas is surprising in light of the prevalence of rural–urban migration as an adaptation strategy in many countries, and the percent of the population living in urban areas, which is higher than 75% in Libya, for example.

Health is another under-represented sector in the literature on observed adaptation, either because there is little adaptation happening, or because it is not being documented [63, 74]. Figure 2 depicts that health reported as a focus less than half as often as “Food, fibre, and other ecosystem products” or “Poverty, livelihoods, and sustainable development”. Epidemics and floods were the most frequent disasters recorded across all 15 countries in this study. In the example of the Democratic Republic of Congo, the country recorded 10 flood disasters and several cholera epidemics over the last 10 years, and their National Adaptation Plan of Action includes the capacity of national meteorological services as a key priority. However, all articles in the GAMI database for DRC were focused on agriculture or migration.

Infrastructure is a similarly under-studied area regarding climate change adaptation in these conflict-affected countries. For example, there were no studies available in the GAMI database that mentioned Israel, Palestine, the West Bank, or Gaza Strip. However, the most funded bilateral projects for the West Bank and Gaza Strip that had climate change adaptation as an objective were related to water infrastructure, especially wastewater treatment and re-use. This is in line with the adaptation policy objectives of the region. In the scholarly literature, articles on wastewater treatment in this location are widely available, but none of them make the link to climate change adaptation. We recommend further research into this discrepancy, to identify the breadth of “adaptation” activities that are not labeled as related to climate change.

The humanitarian sector is also under-represented in the literature on adaptation, even though this sector is strongly represented in adaptation funding flows. In many conflict-affected areas, bilateral funding for adaptation is tagged as a secondary objective of funding for humanitarian objectives. The most prominent examples of this include CAR, South Sudan, and Syria, where the largest category of bilateral aid tagged with adaptation is “Emergency Response.” For example, the largest bilateral project with a climate change adaptation objective for South Sudan is “World Food Programme (WFP) Emergency Operation (EMOP) for the South Sudan Humanitarian Assistance and resilience building programme—Programme Costs”. For Syria, the largest is “ICRC activities in Syria and the region.” Neither country has any research articles on adaptation available in the GAMI database. This represents a large disconnect between the climate change adaptation community and the humanitarian community. While it might be a best practice to include adaptation as a secondary objective of humanitarian programming in these difficult contexts, it is not clear how humanitarian programming is being adjusted in light of climate change, and there are few, if any, links to research.

While research focuses on the links between climate change and conflict, less attention has been paid to how adaptation (and mitigation) responses can lead to conflict, particularly urban violence [78]. More attention is needed concerning applied conflict-sensitive approaches to climate change adaptation and mitigation in conflict-affected countries [60, 69, 78]. Conflict sensitivity means ‘do no harm’ and implies a contribution to peacebuilding [78]. Specific research gaps include: case studies of adaptation activities based on conflict sensitivity; instances where conflict-insensitive adaptation programs have negative outcomes; and assessing the integration of conflict-sensitive thinking and practice within organisations [58, 60, 69].

The gender dimension of climate change impact in agrarian and pastoral communities are well documented in research which has been the main focus of humanitarian and development intervention and funding flows in the last decade. But there is limited literature on the gendered nature of climate adaptation in conflict-affected states [16], and the adaptation responses of men and women in conflict-affected countries is not well captured or understood. Mainstream climate funds are increasingly focusing on gender-responsive mechanisms in climate adaptation finance. For example, the Green Climate Fund has adopted a Gender Action Plan in 2015 intending to mainstream gender considerations in the fund’s scope of beneficiaries to ensure an equal representation of women in the fund’s decision-making processes and as well as to institutionalize gender monitoring and evaluation mechanism for approved projects [25]. This represents a positive trend in recognizing women as dynamic actors in projects and programs related to adaptation in water and agriculture [73] as most of these funding projects are targeted towards these sectors. However, it is not clear how these funding flows will benefit women in conflict-affected areas.

Several geographical locations also emerged as major gaps in this study. Of the 15 countries included, several were the focus of substantial research and funding, with others seeing little to none. Iraq and South Sudan did not have any research articles on climate change adaptation available in the original GAMI database, and these countries receive little multilateral funding for climate change adaptation. There is some bilateral funding to each country that has been tagged as relevant to climate change adaptation.

In some contexts, this analysis at the national level obscures a strong preference for research and investment in peaceful sub-national regions, leaving behind the most conflict-affected locations including refugees who live outside of the conflict area, yet in locations of the hosting countries humanitarian action is prioritized over adaptation. While this is not true of every country studied here, it is a gap that should be investigated further.

There is also potential under-reporting of climate events and their impacts in these conflict-affected countries. For example, only one of the 15 countries reported a heatwave event (Israel) during the period of study, 2010–2019. Further research is needed to better understand the impacts of climate-related disaster events in fragile contexts, and to evaluate the impact of the adaptation investments that are being made in these places. Match et al. [42] agree that climate-related conflict risk could be reduced through addressing known drivers and by incorporating climate into conflict risk reduction assessments through conflict mediation, peacekeeping operations and post-conflict aid and reconstruction efforts. They however emphasize the need to increase understanding of both the effectiveness and the potential adverse side effects of different actions [41]. In the subsequent article, Mach et al. [42] assess the effectiveness and sufficiency of these technologies in managing human security and conflict risks arising from climate change. Policy‐focused analyses that are coupled with experimental research could reveal a full spectrum of drivers and improve policies.

Importantly, the analysis based on GAMI articles is limited by the requirement that activities that qualify as climate adaptation be labeled as such in the academic literature. In some cases, organizations that promote climate adaptation choose to not label it this way for political reasons. Undoubtedly, this study does not capture all of the climate adaptation in conflict affected areas, but rather looks at a sample of climate adaptation activities in order to provide insights about the unique challenges and opportunities in conflict affected countries as well as areas for further exploration.

## Conclusion

While there is ample literature showing that climate change is a risk amplifier for people living in conflict-affected areas, there are large gaps in the information available on the breadth and depth of climate change adaptation activities that are currently happening in these contexts around the world. Several conflict-affected countries and sub-national regions are receiving little to no adaptation research or funding. This lack of documentation could stem from a lack of adaptation activity on the ground as well as a lack of active researchers.

Specific gaps include urban areas, the health sector, infrastructure investments, early warning systems and humanitarian programming for climate adaptation in conflict-affected countries. The climate change adaptation research community seems to be narrowly focused on agriculture and livelihoods and is not engaging with a large portion of the major adaptation investments that are happening in the countries studied here.

Given that people living in conflict-affected areas have some of the highest intersectional vulnerabilities to climate events, greater scholarship on climate change adaptation is critical. We highlight four major priorities for further research. First, a comprehensive review of grey literature on adaptation in these contexts could reveal additional information not included here, as documentation in newspapers, government documents, and development/humanitarian programming documents, and reports in languages other than English can reveal under-studied examples of adaptation. We also suggest a thorough review of climate policies in these countries. Second, greater involvement of local researchers could increase local ownership of the study results and increase focus on priority areas for the country being studied. Involvement of local researchers could be increased if donors and research institutes would require the principle of subsidiarity to be applied. The principle holds that decisions about research and interventions should take place at the most proximate scale of organisation as possible and only when necessary, at a more distant scale. This allows proximate actors with optimal insight of problems to identify research questions, design studies, experience the consequences of their actions, and to revise their theories and inform subsequent action based on local knowledge and feedback, ultimately increasing learning opportunities and building resilience [2].Third, greater involvement of researchers who are not traditionally focused on climate change, such as urban planners and humanitarians, can document the linkages that are being made, or need to be made, in these sectors. Lastly, there is a need for comprehensive studies on the effectiveness and success of adaptation in conflict-affected areas, as this review only documented what has been labeled as adaptation.

While there is evidence of many substantial bilateral aid projects committed for the conflict-affected countries studied here, these projects simply have “climate change adaptation” tagged as a primary or secondary objective. Further research is needed to understand whether and how these projects are adjusting their implementation to achieve adaptation, and to learn from these experiences about the best practices for doing adaptation in a conflict-sensitive manner.

Greater funding flows for these complex contexts are also needed. In particular, funds should include sub-national regions that are experiencing armed conflict, not only the predominantly peaceful regions of a country. Funding should also prioritize the adaptation needs of displaced people, who might no longer be living in the regions of active conflict but are likely to be at high risk during extreme climate events. Adaptation funding from unconventional and mainstream sources would be welcome, such as non-project-based funds for technical advice on conflict sensitivity and space for cross-project and cross-organisational learning [69].

Despite their fragile local context and lack of institutionalized support, people in conflict-affected contexts are confronting climate-related disasters through a variety of coping and adaptation strategies aimed at managing their evolving climate risk. Documenting, supporting, and examining the success of these strategies despite the compounding pressures of conflict, will be critical in the coming years, as climate risks continue to increase and as development trajectories mean that the most vulnerable and impoverished people are primarily found in fragile and conflict-affected areas.

## Data Availability

Open data sources.
